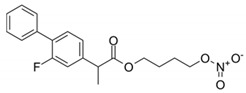# Prospects for the Personalized Multimodal Therapy Approach to Pain Management via Action on NO and NOS

**DOI:** 10.3390/molecules26092431

**Published:** 2021-04-22

**Authors:** Natalia A. Shnayder, Marina M. Petrova, Tatiana E. Popova, Tatiana K. Davidova, Olga P. Bobrova, Vera V. Trefilova, Polina S. Goncharova, Olga V. Balberova, Kirill V. Petrov, Oksana A. Gavrilyuk, Irina A. Soloveva, German V. Medvedev, Regina F. Nasyrova

**Affiliations:** 1 V. M. Bekhterev National Medical Research Center for Neurology and Psychiatry, 192019 Saint-Petersburg, Russia; 2 The Department of Polyclinic Therapy and Family Medicine and Healthy Lifesttyle with a Course of PE, V. F. Voino-Yasenetsky Krasnoyarsk State Medical University, 660022 Krasnoyarsk, Russia; stk99@yandex.ru (M.M.P.); bop_351971@mail.ru (O.P.B.); po.gon4arova@yandex.ru (P.S.G.); kllpetrov@mail.ru (K.V.P.); Oksana.gavrilyuk@mail.ru (O.A.G.); solovieva.irina@inbox.ru (I.A.S.); 3 The Yakutsk Scientific Center for Complex Medicine Problems, 677000 Yakutsk, Russia; tata2504@yandex.ru (T.E.P.); ynckmp@yandex.ru (T.K.D.); 4 The Hospital for War Veterans, 193079 Saint-Petersburg, Russia; 5 Olympic Sports Research Institute, The Ural State University of Physical Culture, 454091 Chelyabinsk, Russia; olga-balberova@mail.ru; 6 R. R. Vreden National Medical Research Center for Traumatology and Orthopedics, 195427 Saint-Petersburg, Russia; dr.madvedev.g@yandex.ru

**Keywords:** pain, neuropathic pain, inflammatory pain, posttraumatic pain, intervertebral disc, degeneration, facet joints arthrosis, myofascial syndrome, back pain, polyneuropathy, trauma, nitric oxide (NO), nitric oxide synthase (NOS), gene, genetics, genetic predisposition

## Abstract

Chronic pain syndromes are an important medical problem generated by various molecular, genetic, and pathophysiologic mechanisms. Back pain, neuropathic pain, and posttraumatic pain are the most important pathological processes associated with chronic pain in adults. Standard approaches to the treatment of them do not solve the problem of pain chronicity. This is the reason for the search for new personalized strategies for the prevention and treatment of chronic pain. The nitric oxide (NO) system can play one of the key roles in the development of peripheral pain and its chronicity. The purpose of the study is to review publications devoted to changes in the NO system in patients with peripheral chronical pain syndromes. We have carried out a search for the articles published in e-Library, PubMed, Oxford Press, Clinical Case, Springer, Elsevier, and Google Scholar databases. The search was carried out using keywords and their combinations. The role of NO and NO synthases (NOS) isoforms in peripheral pain development and chronicity was demonstrated primarily from animal models to humans. The most studied is the neuronal NOS (nNOS). The role of inducible NOS (iNOS) and endothelial NOS (eNOS) is still under investigation. Associative genetic studies have shown that single nucleotide variants (SNVs) of *NOS1*, *NOS2*, and *NOS3* genes encoding nNOS, iNOS, and eNOS may be associated with acute and chronic peripheral pain. Prospects for the use of NOS inhibitors to modulate the effect of drugs used to treat peripheral pain syndrome are discussed. Associative genetic studies of SNVs *NOS1*, *NOS2*, and *NOS3* genes are important for understanding genetic predictors of peripheral pain chronicity and development of new personalized pharmacotherapy strategies.

## 1. Introduction

Chronic pain syndromes are an important medical problem generated by various molecular, genetic, and pathophysiologic mechanisms. Back pain, neuropathic pain, and posttraumatic pain are the most important pathological processes associated with chronic pain in adults. Standard approaches to the treatment of these chronic pain syndromes do not solve the problem of pain chronicity. This is the reason for the search for new personalized strategies for the prevention and treatment of chronic pain. The nitric oxide (NO) system can play a key role in the development of peripheral pain and its chronicity. The cost of treating chronic pain is high worldwide, regardless of where the patient lives. Most new episodes of acute peripheral pain syndromes are accompanied by a quick recovery. However, chronic peripheral pain syndromes leading to temporary disability are common, and in some patients, they become permanent, leading to high rates of disability. The socio-economic significance of these disorders results from their prevalence among adult patients and associated significant economic losses (insurance payments and decrease in labor efficiency during the period remission) [1]. Specifically, chronic peripheral pain syndromes are reported to lead to significant economic losses in the US [2]. In the US, 20 million people suffer from chronic pain, the economic damage being 50 billion dollars per year, and compensation payments to patients with spinal diseases due to temporary working capacity amounts to 200 million dollars per year. The cost of the back pain treatment is 2–3 times higher than the cost of the treatment of cancer patients [3]. In Europe, the socio-economic burden of back pain is also very high. A minority of patients have recurrence to secondary or tertiary care because of severe and long-lasting complaints. This subgroup may account for a major part of disability and costs yet could potentially gain most from treatment [4].

**Figure 1:**
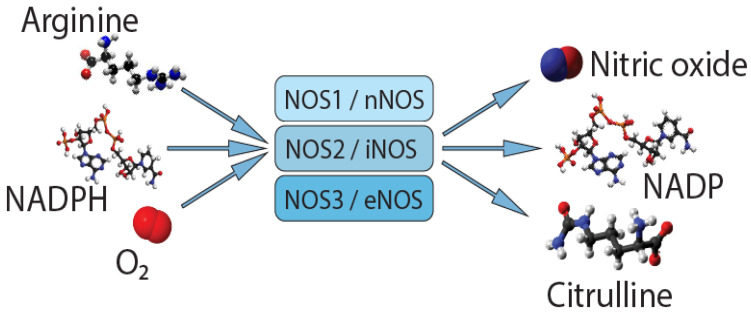
Synthesis of nitric oxide.

**Figure 2:**
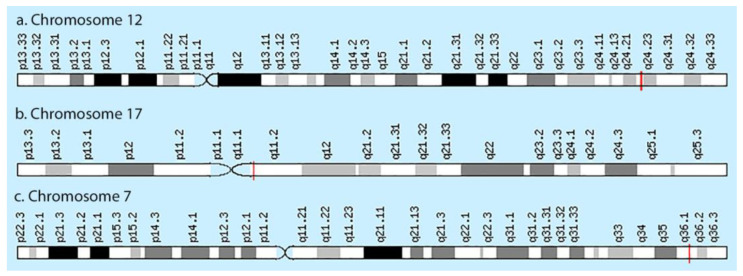
Localization of *NOS1* (**a**), *NOS2* (**b**), and *NOS3* (**c**) genes.

The NO system can play one of the key roles in the development of peripheral pain syndrome and its chronicity. NO is involved in numerous physiological processes in the peripheral and central nervous system. It is produced intracellularly by the catabolism of L-arginine to L-citrulline by NO synthase enzyme (NOS), which is present in three isoforms, including neuronal (nNOS), endothelial (eNOS), and inducible (iNOS) isoforms (Figure 1). The role that NO plays in pain is not simple, since it may show pro- or antinociceptive effects depending on the circumstances. The majority of data, in preclinical studies, support a pronociceptive role of NO at the spinal level. Yet, other studies show inconsistent results [5]. *NOS1*, *NOS2*, and *NOS3* genes (Figure 2) are implicated in the production of nNOS, iNOS, and eNOS [6].

The purpose of the study is to review the publications devoted to changes in the NO system in patients with peripheral chronic pain syndromes.

## 2. Materials

We carried out a search for full-text articles published in e-Library, PubMed, Oxford Press, Clinical Case, Springer, Elsevier, and Google Scholar databases. The search was carried out using keywords and their combinations, including pain, neuropathic pain, inflammatory pain, posttraumatic pain, intervertebral disc, degeneration, facet joints arthrosis, myofascial syndrome, back pain, polyneuropathy, trauma, nitric oxide (NO), nitric oxide synthase (NOS), gene, genetics, and genetic predisposition. The search depth was 20 years (2001–2021). In addition, articles of historical interest have been included in the review. Despite an extensive search, it is possible that we might have missed some studies published in recent years.

## 3. Results

### 3.1. Back Pain

#### 3.1.1. The Role of NO and *NOSs* in the Development of Back Pain

Back pain is a progressive and debilitating disease with multifactorial causes (intervertebral disc degeneration (IVDD), arthrosis of the facet joints, and muscular tonic syndrome, etc.). However, the mechanisms of chronic back pain are poorly understood. At the same time, IVDD and arthrosis of the facet joints are considered to be the two leading causes of this disorder [7].

NO is an oxygen-free radical which is involved in a variety of physiological and pathological events. NO concentration may increase in the perifacetal region, and perifacetal NO levels in patients with chronic pain in the back were higher compared to healthy people. Brisby H. et al. (2007) showed that patients with chronic back pain have three-fold higher level of NO in the perifacetal region compared to the healthy controls (1.66 ± 1.39 vs. 0.46 ± 0.37 nM, *p* = 0.007). However, the authors did not find an association between NO level and pain duration or pain level, which was rated on the visual analogue scale (VAS). Research has shown that higher NO level in the perifacetal region may be the biomarker of chronic low back pain in patients with facet joint arthrosis. The findings of higher NO levels in the perifacetal region in chronic low back patients compared to healthy controls indicate that the degenerative process of the joints may cause increased NO production. Patients that responded to corticosteroid/local anesthetic infiltration had higher NO level in the perifacetal region compared to patients without response [8].

The role of different NOS isoforms in low back pain process is derived primarily from animal models [9]. While nNOS is mainly observed at the spinal level or in neuropathic pain models, iNOS is upregulated in inflamed tissues [10] and is involved in the development of hyperalgesia in inflammatory and neuropathic pain models [11]. Jensen L. et al. (2015) investigated the role of nNOS in the mechanism of chronic muscle pain development on the example of trapezius myalgia in 42 women with clinically diagnosed neck pain. The authors showed that sarcolemmal nNOS expression is irregular and absent from selected fibers in the trapezius muscle. Moreover, they found an increase in sarcoplasm-localized nNOS in women with trapezius myalgia, which was essentially normalized by 10 weeks of specific strength training. Abnormalities in nNOS expression show a potential of predicting the progression of muscle damage and pain and correcting the dislocation of nNOS may prove essential in treatment of work-related muscle pain [12]. Kohyama K. et al. (2000) showed that changes in the level of NO can be associated with intervertebral disk due to the induction of apoptosis of intervertebral disk cells [13]. Oxidative stress is a cellular state with an increased level of reactive oxygen species (ROS) that is caused by an imbalance of generation and removal of these species. Singlet oxygen, superoxide anions, hydroxyl radicals, and NO are examples of ROS synthesized in intervertebral disk cells [14]. In intervertebral disk tissues, oxidative stress may initiate or participate in matrix destruction and cell apoptosis, which ultimately result in disk degeneration [15]. It has been shown that the increase of exogenous NO may promote cell apoptosis and suppress proteoglycan synthesis in cultured disk cells. NO has been demonstrated to participate in the IVDD induced by mechanical stress or interleukin (IL)-1 [13,16,17,18,19].

Liu G.Z. et al. (2001) and Rannou F. et al. (2003) demonstrated that NO mediates the change of proteoglycan synthesis in the annulus fibrosus cells of the human intervertebral disc [17,18].

#### 3.1.2. Association of SNVs of *NOSs* Family Genes with Back Pain 

In order to determine the role of SNP genes *NOSs* in herniation of the lumbar intervertebral disk in humans, a case-control study was carried out with the participation of 179 adult individuals, according to the results of which SNP *NOS3* rs2070744 (−786 T/C) and *NOS2* rs1060826 (22 G/A) were more common among the control group than in patients with symptoms of hernia of the lumbar intervertebral disk, including those with pain syndrome, which indicates their possible protective role against hernia of the lumbar intervertebral disk. Genotyping of these SNPs can be useful in identifying individuals with an increased lifetime risk of disc herniation, in whom measures to prevent disc herniation in the lumbar spine can be applied. This study also examined polymorphism *NOS3* 4a4b (27-bp variable number of tandem repeat (VNTR) polymorphism in intron 4); however, when comparing the genotypic and allelic frequencies of this SNP genotyped in patients with hernia of the lumbar intervertebral disk and in the control group, no significant differences were observed [20]. In a later study among the patients with the IVDD in a young Korean population, the disc degeneration severity score was analyzed according to the genotypes of VEGF and eNOS. The results showed that the frequencies of *NOS3* 786TC + CC (21.6%) and 894GT + TT (27.5%) were higher in the patients compared with controls. However, no statistically significant differences between patients and controls were found [21].

Despite the fact that the expression of nNOS, which is encoded by the *NOS1* gene, is shown in the structures of the intervertebral discs [7] and in skeletal muscles [22], the prognostic role of the *NOS1* gene of SNVs in the development of back pain in patients with IVDD, facet joints arthrosis, and muscle-tonic syndromes has not yet been studied.

#### 3.1.3. Prospects for the Use of NOS Inhibitors to Modulate the Effect of Drugs Used to Treat Back Pain

NSAIDs are among the most prescribed medications for the patients with low back pain, yet their efficacy is compromised by a ceiling analgesic effect. The inhibitory effect of NSAIDs on NO production has been reported in many studies both clinically [23] and experimentally [24,25] and the involvement of the NO-cGMP pathway in the antinociceptive effects of NSAIDs has been suggested in several experimental studies [26,27].

Hamza M. et al. (2010) studied the effect of ketorolac on NO production and *NOSs* gene expression. The authors showed significantly higher levels of NO at the first 100 min compared to the last 80 min in the placebo-treated group. In the patients treated with ketorolac, NO level gradually decreased over the first 60 min but was similar to placebo over the later 100–180 min, with no significant change in NO level over time. The levels of NO were negatively correlated to pain intensity scores. While the gene expression of iNOS and eNOS were not upregulated, 3 h after surgery, nNOS was downregulated in both treatment groups and *NOS3* gene expression was significantly lower in the ketorolac group compared to the placebo group [28].

Intradermal administration of the NOS substrate L-Arginine or the NO donor SIN-1, both of which elevate NO levels, cause a dose-dependent mechanical hyperalgesia [29]. However, the intracutaneous injection of NO in healthy volunteers evokes pain in a dose-dependent manner [30], while transdermal application of the NO donor glyceryl trinitrate improved pain in patients with shoulder pain syndrome [31].

The research by Fujioka Y. et al. (2016) showed that gene *NOS1* expression, associated with inflammatory and neuropathic pain, was upregulated at days 1 and 3 in the dorsal root ganglion following disc puncture; it might serve as a therapeutic target for lumbar disc herniation. It is not known whether inhibitors of *NOS1*, such as L-NG-Nitroarginine methyl ester (L-NAME), are useful for low back pain and sciatica [32].

Kohyama K. et al. (2000), studied the biological understanding of IVDD. The authors showed a change in iNOS expression in intervertebral disk cell culture obtained from the surgical specimen of the patients with lumbar IVDD with herniation. Suppression of 3H-Thymidine incorporation and DNA fragmentation in the disc cells were promoted by treatment of 100 microM NOC-18. These results suggest that the use of modulators of NO level and NOS activity is important for slowing down the rate of IVDD development [13].

Yang X. et al. (2014) showed that the use of an antioxidant nanofullerol prevents IVDD. The authors hypothesize that antioxidants are potentially good therapeutic candidates to treat IVDD, because of their ability to scavenge ROS that leads to the injury of cellular functions in intervertebral disk tissues. Fullerenes are extremely powerful antioxidants with unique nanostructures, and superior to conventional antioxidants due to their long-lasting activity and excellent cell membrane-penetrating ability. In this study, Yang X. et al. (2014) found that fullerol (a polyhydroxylated derivative of fullerene) had a preventive effect on IVDD, highlighting its potential use as a therapeutic agent for the treatment of lower-back pain. Nanofullerol reduced the cytotoxicity and ROS of hydrogen peroxide-treated human nucleus pulposus cells and counteracted in vivo IVD in a rabbit annulus-puncture model [14].

Nerlich A.G. et al. (1997) reported that carboxymethyl lysine (CML) is associated with ROS accumulation in the nucleus pulposus tissue, and that CML was closely related to IVDD [33]. The authors indicated that CML activated a receptor of advanced glycation end products (RAGE)-nuclear factor (NF)-κB system in aging and IVDD. The activation of RAGE-NFκB promoted the gene expression of *NOSs* and metalloproteases (MMPs) [15]. It is not clear whether nanofullerol defends IVDD by modifying CML and its signaling pathway, and this is currently under investigation [14].

Castania, V. et al. (2017) analyzed the potential role of nNOS modulation in the tail needle puncture model of IVDD. Male Wistar rats were submitted to percutaneous disk puncture with a 21-gauge needle of coccygeal vertebras. The selective nNOS pharmacological inhibitor N (ω)-propyl-L-arginine (NPLA) or a nNOS-target siRNA (siRNAnNOShum_4400) was injected immediately after the intervertebral disk puncture with a 30-gauge needle. Signs of IVDD were analyzed by in vivo MRI and histological score. The authors found that intact intervertebral disks express low levels of nNOS mRNA. Disk injury caused a four-fold increase in nNOS mRNA content at 5 h post disk lesion. However, NPLA or nNOS-target siRNA slight mitigate IVDD progress. Further studies would disclose the nNOS role and its potential therapeutical value in IVDD [7]. Current therapies for IVDD and facetal joints arthrosis with acute or chronical back pain may include nonsurgical treatment with NO modalities [16,34] and include presurgical and postsurgical periods in the patients with intervertebral disk herniation [35,36]. However, neither nonsurgical nor surgical therapy restore functional native intervertebral disk tissue, or regenerate the degenerated intervertebral disk tissue, resulting in chronic inflammatory back pain. In recent years, numerous studies have demonstrated that oxidative stress and local NO levels play an important role in the initiation and progression of IVDD [19,33,37,38] and back pain [8,11].

### 3.2. Neuropathic Pain

#### 3.2.1. Role of NO and *NOSs* in the Development of Neuropathic Pain

Polyneuropathies (PNP) are a large group of the peripheral neuro system diseases, characterized by symmetrical diffuse lesions of peripheral nerve fibers [39,40,41]. The unit of damage in PNP is not individual nerves, but fibers that make up various peripheral nerves, the probability of damage to which depends on their length, caliber, antigenic composition, metabolic rate, etc. Among the most common forms of PNP are diabetic PNP, chronic inflammatory demyelinating polyneuropathy (CIDP), alcoholic, hereditary Charcot-Marie-Tooth neuropathy, paraneoplastic PNP, and Guillain-Barret syndrome [42,43,44,45]. There are sensory, motor, sensorimotor, and autonomic polyneuropathies, depending on the predominant involvement of the type of nerve fibers in the pathological process [46,47,48]. One of the leading clinical symptoms of sensory PNP is neuropathic pain [49]. NO plays a huge role in the development of neuropathic pain [50]. nNOS is mainly found in the nervous system, and is required for the transmission of neuronal signals, while eNOS is localized in the endothelium and required for vasodilation and blood pressure control [51]. NOS is widely involved in the formation of neuropathic and nociceptive pain [52,53,54,55,56]. The main role of iNOS in human physiology is the elimination of invading pathogens by participating in the development of the inflammatory response and the responses of the immune system [57]. Neuronal NO plays an important role in nociception and opioid action in the central and peripheral nervous system. The importance of the NMDA/NO cascade in opioid tolerance has been extensively studied. The ability of NOS inhibitors to block morphine tolerance has been established; they also directly affect morphine pain relief [58,59]. In experimental studies, NO has also been associated with PNP, which has been proven in experimental models: injection of formalin into the hind paw, surgical ligation of the spinal nerve, or ligation of the sciatic nerve in animals [60,61]. Thus, formalin injection into the hind paw in rats increases the number of neurons containing NOS in the L4-L5 region of the dorsal horn of the spinal cord [62]. Peripheral nerve damage can lead to central sensitization of neurons in the posterior horns of the spinal cord by activating nociceptive afferent cells, followed by activation of NMDA receptors and NO production in spinal cord cells [63,64,65,66,67].

An association of nNOS expression in the ventromedial prefrontal cortex with anxiety in chronic neuropathic pain has been established [68]. Various studies have shown that iNOS (both centrally and peripherally expressed) may play a role in the development and sensation of inflammatory and neuropathic pain, as demonstrated in animal models of neuropathic pain responses [69]. Meller S.T. et al. (1994) found that lipopolysaccharide (LPS) and cytokines induce thermal hyperalgesia (hypersensitivity to pain), possibly mediated by the induction of iNOS in glial cells of the spinal cord [70]. Thus, the development of iNOS inhibiting therapeutic agents in the treatment of pain is very promising.

#### 3.2.2. Association of SNVs of *NOSs* Family Genes with Neuropathic Pain

The participants in the study “Epidemiology of Diabetic Complications” in Pittsburgh, Pennsylvania, first observed in 1986–1988, were divided into subgroups corresponding to whether they had experienced diabetes mellitus complication; 15 candidate genes were compared between the groups. The carriage of homozygous GG genotype (Glu298Asp, E298D) of the eNOS gene increased five-fold the risk of confirmed distal symmetric PNP for diabetic patients (*p* < 0.05). The SNV G894T of eNOS gene may be important for the neuropathic pain development in the patients with PNP. Costacou T. et al. (2006) found that the allele T of this SNV was associated with neuropathic pain in the diabetic PNP patients [71]. Zotova E.V. et al. (2005) studied the alleles and genotypes frequency of polymorphic markers of genes *NOS1*, *NOS2*, and *NOS3* in Russian patients with diabetic PNP. The authors did not find significant differences in the alleles and genotype frequency of the polymorphic markers (CA)n of the *NOS1* gene, (CCTTT)n of the *NOS2* gene, and ecNOS4a/4b and Glu298Asp of the *NOS3* gene. Only the (CCTTT)n marker of the *NOS2* gene was associated with diabetic PNP and neuropathic pain development [72].

#### 3.2.3. Prospects for the Use of NOS Inhibitors to Modulate the Effect of Drugs Used to Treat Neuropathic Pain 

Considering the above, the use of NOS inhibitors in the treatment of neuropathic pain is very promising. Systemic non-selective NOS inhibitor N-nitro-L-arginine (NOArg) prevents and changes morphine tolerance [59]. Today, the use of iNOS inhibitors is proposed, which directly affect the enzyme. Some of the drugs under development were rejected due to low selectivity and the development of adverse events. For the neuropathic pain treatment, amino acid amidine proved to be effective, which showed an analgesic effect and entered phase II clinical trials, which were discontinued in 2012 [11]. Optimization of bis-isothiourea made it possible to obtain a highly selective inhibitor of iNOS, which had a good effect on neuropathic pain in experimental animals, which was confirmed by an increased level of analgesic cytokines [73].

4-Methylaminopyridine is a non-selective iNOS inhibitor that is active in rodent endotoxemia and pain models, although the dose required for pain relief is lower than the dose required for efficacy in an endotoxic shock model, suggesting some of its analgesic properties are likely to be inhibition of nNOS [74]. 

The second mechanism on which the development of inhibitors is focused is the inhibition of the formation of the active iNOS dimer [75]. Thus, pyrimidinylimidazoles and homoproline were shown to be effective in arthritis due to the binding of iNOS monomers [76,77], but this drug was not studied in PNP patients with neuropathic pain.

In 2009, quinolinone chit was identified, and its new analogs showed efficacy against pain behavior in mice when formalin was administered [78]. Their use is limited by their short half-life. The classic indication for the use of iNOS inhibitors is sepsis, but the presence of serious side effects limits the use of these drugs in neuropathic pain treatment. Antinociceptive use of iNOS inhibitors in humans has not demonstrated the desired effect [79]. Rocha P.A. et al. (2020) described a higher number of nNOS and iNOS-positive neurons in the spinal cord and posterior horns. The authors showed that the use of selective inhibitors of nNOS and iNOS may be effective in relieving neuropathic pain [80]. The effects on nNOS activity are good prospects in the treatment of acute and chronic neuropathic pain in PNP patients [81].

### 3.3. Post-Traumatic Pain

#### 3.3.1. The Role of NO and *NOSs* in the Development of Pain after Muscle, Tendon, and Joint Sports Injuries

NO is one of the most powerful and unconventional signaling molecules in the body. NO can be synthesized from arginine in a reaction catalyzed by four splicing variants of neuronal nitric oxide synthase (nNOS) (α, β, γ, and μ). There is a fifth variant called nNOS-2, but it is unclear whether it can synthesize NO. nNOS splice variants are highly expressed in skeletal muscle. Skeletal muscle expresses two forms of the nNOS compound: nNOSß and nNOSμ. nNOSμ is the main enzymatic source of NO in skeletal muscle [82]. Because of its widespread expression in skeletal muscle, accounting for ≈40% of body weight, nNOS_M_ is responsible for the largest proportion of NO generated by the NOS enzyme in the body. It is important to note that NO from nNOS can be stored as nitrates, making skeletal muscles the largest potential storage of NO in the body [83]. Thus, skeletal muscle is a powerful model system for understanding the function of the nNOS enzyme and the biology of NO [84]. The greatest interest in nNOS function in skeletal muscles arose from the discovery that the expression and sarcolemmal localization of nNOSμ were impaired in the skeletal muscles of individuals with Duchenne and Becker muscular dystrophy [85].

The diversity of nNOS transcripts can be explained by alternative splicing of *NOS1* gene exons in an open reading frame. This plays an important role in establishing a skeletal muscle-specific expression profile of the nNOS splicing variant. An example of this is the induction of nNOSµ expression during muscle cell differentiation. Immature primary myoblasts and myotubes express nNOSa. It remains unclear whether nNOSß is expressed in immature muscle cells. Thus, nNOSμ is expressed when muscle cells differentiate into myotubes, which can express both nNOSa and nNOSμ. However, fully differentiated myofibers in musculoskeletal tissue express only nNOSμ, as well as nNOSß [82]. Indeed, nNOSμ is the predominant form of splicing, accounting for 95% of total nNOS expression and 85% of NOS activity in mature skeletal muscles [86]. Thus, nNOSß is a small but, as we will see, important source of NO, while the expression and function of nNOSa may be relevant for non-fully differentiated immature muscle cells.

The importance of considering isoform-specific nNOS function in skeletal muscle is further emphasized by the discovery of nNOSß localized in Golgi complex. This is convincing proof of the unique role of nNOSμ and nNOSβ in the regulation of muscle structure, strength, fatigue resistance, and blood supply to the muscles [87].

NO has been shown to play an important role in both physiological and pathological conditions in athletes. Chronic diseases of the muscles and tendons are extremely common in sports practice and represent a serious clinical problem. NO is a powerful regulator and stimulator of biological processes, including degeneration and muscle and tendon healing. It is also involved in the reaction to mechanical stimuli in various tissues [88].

Depending on the source of production, NO can play a protective or negative role in muscle and tendon repair. Although NO is a highly diffusible gas, signal transmission through the two constitutively expressed isoenzymes (nNOS and eNOS) is separated, and each isoform modulates physiological functions differently [89]. There is evidence that the iNOS isoform is not constitutively expressed, but its expression is induced during the inflammatory response. However, in studies by Bokhari AR et al., it was shown that after tendon injury, NO is induced by all three isoforms of NOS and that NOS activity is increased in tendinopathy [90].

In normal uninjured tendons, NOS activity is very low. Lin J. et al. (2001) found a cell-specific time pattern for mRNA and protein for all three NOS isoforms after Achilles tendon injury. iNOS was maximal on 4th day in macrophages and fibroblasts. eNOS was maximal on 4th day in endothelial cells and fibroblasts. nNOS expression gradually increased to 21st day and was found only in fibroblasts. The authors hypothesized that all three NOS isoforms are expressed by fibroblasts in a coordinated time sequence during tendon healing. The consistent pattern of NOS expression in healing fibroblasts suggests that each NOS isoform may play a different role in the healing process and provides opportunities for changing tendon healing in clinical settings [91].

In the studies by Szomor Z.L. et al. (2006), temporal and differential expression of NOS isoforms has been demonstrated in acute tendon injury healing. Because of this, the authors suggested that different patterns of NOS expression may have different biological functions. Significant stress on the tendon-ligamentous apparatus in athletes, especially runners, skiers, and speed skaters, can lead to differential activation of NOS, especially iNOS. Szomor Z.L. et al. (2006) used an animal model of excessive stress on the tendons of the supraspinatus muscle, consisting of running on a treadmill. After 4 weeks of regular exercise, RNA was extracted, and competitive reverse transcription and polymerase chain reaction (RT-PCR) was performed to determine the expression levels of *NOSs* isoforms in the supraspinatus tendons. mRNA expression of all three *NOSs* isoforms increased in the supraspinatus tendons as a result of excessive exertion. A statistically significant increase in eNOS expression was found. Increase in mRNA and nNOS expression was not statistically significant compared to the control group [88].

Damage to cartilage tissue can be caused by both biochemical and mechanical factors. Abramson S.B. (2008) [92] suggested that NO and its redox derivatives appear to perform a number of different functions in both healthy and injured joints. Until recently, NO was considered a catabolic factor responsible for aggravating cartilage disease processes by mediating pro-inflammatory cytokines expression, inhibiting collagen and proteoglycans synthesis, and inducing apoptosis. Mazzetti I. et al. (2001) indicated that high local concentrations of NO negatively affect chondrocyte’s function, inhibiting collagen matrix components and proteoglycans synthesis, activating metalloproteinases, reducing proinflammatory cytokine receptor antagonist-interleukin 1β(IL-1β) expression, inhibiting proliferation, and provoking chondrocytes apoptosis by activating caspase-3 and tyrosine kinases [93]. However, other studies show that NO and its redox derivatives can also have a protective effect on cartilage [92].

A simple view of NO signaling is that after its synthesis, NO diffuses out of the cell and activates soluble cyclic guanylate cyclase (sGC) to produce activated cGMP from guanosine triphosphate (GTP). cGMP then specifically binds to target proteins, which include protein kinases, phosphodiesterases, and cyclic nuclear ion channels, causing a number of biological effects. However, studies have shown that NO inhibits the synthesis of both proteoglycans and collagen. In human alginate cultures of articular chondrocytes, endogenously produced NO inhibited proteoglycans synthesis from both the superficial and deep cartilage zones [94]. However, in the presence of the NOS inhibitor NG-monomethyl-L-arginine (L-NMA), proteoglycan synthesis suppression was completely reversed in deep chondrocytes, but only partially reversed in the surface zones, suggesting that NO may be a protective agent in response to proteoglycan catabolism [95].

It is not possible to determine whether the increase in procollagen mRNA was caused by an increase in NO signaling or by another mechanism. It is clear that NO signaling has opposite effects under different conditions, and it is also clear that more research is needed to elucidate the mechanism of these different cartilage responses to an increase in local NO levels after sports injuries.

#### 3.3.2. Association of SNVs of *NOSs* Family Genes with Pain after Muscle, Tendon, and Joint Sports Injuries

Some genetic markers located in genes encoding structural proteins, extracellular proteinases, and signaling molecules are associated with damage to muscles, tendons, and joints. The analysis of the literature data indicates that the genetic contribution to these lesions is polygenic and that several biological pathways are involved. The polygenic nature of muscle, tendon, and joint injuries is not in doubt, since many internal risk factors are determined by both genetic factors and epigenetic environmental factors, including hard physical loadings.

SNP G894T (Glu298Asp, rs1799983) is associated with several human health phenotypes and/or the body’s response to exercise. Saunders C.J. et al. (2006) found a tendency to a higher frequency of carrying the allele 894G (Glu298) of the *NOS3* gene in combination with the homozygous genotype -9/-9 of the *BDKRB2* gene in elite triathletes compared to the control group (non-athletes) [96].

Brooks C. et al. (2020) conducted a genetic association study of two SNPs (rs2779249 C/A; rs2248814 A/G) of the *NOS2* gene encoding the iNOS enzyme as Achilles tendinopathy predictors in British population. The authors showed that heterozygous genotype C/A rs779249 was protective against Achilles tendon pathology compared to the control group (*p* = 0.009). However, a specific sex-adjusted statistical analysis showed that this protective effect was statistically significant in men (*p* = 0.016), but not in women [97].

The studied SNP rs2248814 alleles did not significantly affect the risk of Achilles tendinopathy or other Achilles tendon pathologies. This study may be important for future genetic studies of modified risk factors for Achilles tendon injuries in athletes. In addition, these studies confirm the important role of NO in the health and/or tendons degradation, including after excessive physical loadings.

#### 3.3.3. Prospects for the Use of NOS Inhibitors to Modulate the Effect of Drugs Used to Treat Posttraumatic Pain

Increasing collagen synthesis is very important for improving muscle, joint, and tendon strength and integrity. This process is especially important when tendons are healing, since the collagen-containing type I fibril is the main element responsible for the structure stabilization and mechanical properties of this tissue. Treatment of tendinopathy is a complex process controlled by many factors. Evidence is emerging for an important role in NO recovery in sports injuries treatment. Being one of the smallest molecules in nature, NO quickly diffuses through the membranes and biological structures inside the cell and its surrounding matrix. In biological fluids, the half-life of NO is only a few seconds, because it reacts easily with other atoms or molecules. NO has a high affinity for heme and non-heme iron, sulfide or thiol groups, superoxide anion, and molar oxygen. In the presence of oxygen, noradrenaline is metabolized to nitrite (NO_2_), which is then oxidized by oxyhemoglobin in blood to nitrate (NO_3_). Under cultivation conditions, nitrite and nitrate are stable metabolites of NO. Nitrite and nitrate are often measured as marker moles for NO production.

Systemic inhibition of nNOS activity has disastrous consequences for skeletal muscles [98]. However, it is not clear whether the decrease in nNOS activity in skeletal muscles is the only cause of the development and chronicity of pain syndrome after sports injuries. At the same time, it has been shown that the nNOS activity loss in tissues outside the muscles can also indirectly contribute to post-traumatic skeletal muscle dysfunction. Despite this, nNOSß may be an important regulator of skeletal muscle function. The sarcolemmal pool of nNOSμ has received the most attention with strong evidence for its role in vasomodulation and possibly muscle atrophy, including after excessive loading and/or traumatization [99]. Accordingly, preservation of nNOSm expression can protect skeletal muscles from atrophy [100]. Strong evidence for the distinct role of sarcolemmal nNOSm has been obtained from studies of the mechanisms regulating blood flow in contracting human skeletal muscles [87].

Barouch L.A. et al. (2002) [89] found increased NOS activity in fibroblasts of damaged human tendons, which plays a role in the processes of repair (healing of tissues damaged as an injury result). In animal models, competitive inhibition of NOS resulted in reduced tendon healing, whereas the addition of NO resulted in enhanced tendon healing. In cultured human tendon fibroblasts, chemical addition of NO and transfection with adenovirus resulted in enhanced collagen synthesis. The authors conducted three randomized, double-blind clinical trials, demonstrating a significant positive effect of NO treatment on clinical symptoms and physical activity in patients with Achilles tendinopathy, tennis elbow, and supraspinatus tendinitis. NO was delivered via glycerol trinitrate (GTN) patches. A three-year follow-up demonstrated significant long-term efficacy of GTN patches in the treatment of non-insertional Achilles tendinopathy [90].

Similar results were obtained in the study by Murrell G.A. et al. (2007), who in a series of experiments showed that NO is produced by all *NOSs* isoforms during tendon healing and that it plays a crucial positive role in restoring tendon function [101]. In the damaged human tendons, *NOSs* activity was expressed in the sequential temporal activation of different isoforms expression. In healing Achilles tendon fibroblasts, the first isoform expressed was eNOS, followed by iNOS and then nNOS. In other studies, conducted by the same authors [101] on an animal model, it was found that systemic suppression of *NOSs* activity reduced the cross-sectional area and mechanical properties of the Achilles tendons. The release of NO by HCT-1026, a nitrate derivative of flurbiprofen, improved the healing of the Achilles tendon. The addition of NO to cultured human tendon cells by chemical means and through adenovirus transfection enhances collagen synthesis, suggesting that matrix synthesis may be one of the mechanisms for the positive effect of NO on tendon healing.

Murrell G.A. et al. (2007) showed that the local use of applications with GTN compared with placebo in athletes with tendinopathy of different localization has a positive clinical effect [101]. Thus, in patients with tennis elbow, the clinical effect of reducing pain and increasing physical activity was achieved in 81% of cases (in the NO group) compared to 60% of patients with standard rehabilitation, without GTN use [102]. In the NO-GTN group, patients with Achilles tendonitis had improved foot function testing, reduced pain, and increased daily physical activity, which was shown in 78% of cases compared to 49% of cases in the control group (standard rehabilitation without the use of GTN); the differences were significant (*p* = 0.001) [102].

In addition, the use of NO significantly reduced shoulder pain with an increase in the volume of active movements during abduction, flexion, and external rotation in patients with supraspinal tendinopathy in 46% of cases compared to 24% of cases in the control group (*p* = 0.007) [102].

Studies by Xia W. et al. (2006) [103] confirmed that NOS increases during tendon healing and inhibition of NOS leads to a significant reduction in the recovery time of the Achilles tendon. It was also noted that local NO use reduced pain and increased functional mobility in patients with tendinosis of the Achilles tendon, the extensor mechanism in the elbow and shoulder.

## 4. Discussion

Peripheral pain is a common clinical syndrome that seriously affects the quality of life and work capacity of patients. NO induces sensitization and chronicity of pain and causes apoptosis of neurons in inflammatory pain [104]. However, the role of NO and NOS in pain syndromes in patients with neurological disorders continues to be debated. Recent studies have shown that NOS enzymes play an important role in the development and chronicity of neuropathic, inflammatory, and post-traumatic pain. On the one hand, it is believed that afferent neurons in the spinal cord release glutamate to activate NMDA receptors, causing Ca^2+^ influx and NOS activation in neuropathic pain. NO promotes the release of glutamate and increases pain [105]. On the other hand, NO and cGMP can activate target molecules, including protein kinase G and various types of potassium channels, resulting in analgesic effects. Many studies have shown a significant role for NO in acute and chronic pain in neurological diseases (spinal cord diseases; genetic, inflammatory, autoimmune and post-traumatic neuropathies; neurodegenerative disease; etc.). However, the conflicting results of these studies highlight the changing role of NO in the development of pain syndromes. It is known that NO is involved in the modulation of peripheral and central nervous nociceptive pathways, especially in the spinal cord [106]. Alteration of peripheral neurons predetermines structural changes in the spinal cord, taking into account the mediator role of NO in the regulation of nociceptive pathways of the dorsal horn of the spinal cord [107]. The role of NO as a “retrograde transmitter” with easy transmembrane penetration into neurons is to activate NO production in the postsynaptic neuron and its diffusion into the presynaptic neuron in order to modulate excitability and enhance synaptic transmission [108]. Regulation of NO is a complex process, and all *NOSs* enzymes (nNOS, iNOS, and eNOS) are catalysts for the rate of NO synthesis in health and disease. Thus, in the norm, iNOS is mainly absent in the nervous tissue, but it increases in patients with chronic pain compared to healthy people. The various forms of NOS differ not only in their localization, but also in their function. Two calcium-dependent constitutive *NOSs* (eNOS and nNOS) generate small amounts of NO, while iNOS production does not depend on intracellular calcium concentrations and generates damaging levels of NO for cells and microorganisms [109]. These enzymes are different proteins encoded by different genes (*NOS1*, *NOS2*, *NOS3*). However, they have the same genomic structure and similar catalytic activity [10]. It is known that iNOS is activated in inflamed tissue [10] and is involved in the development of hypersensitivity to pain in inflammatory and neuropathic animal models of pain [11]. The use of iNOS inhibitors can block inflammatory and neuropathic pain syndromes.

NO generated by eNOS modulates the inflammatory mechanism of pain syndrome due to adhesion of leukocytes, and also participates in the regulation of vascular tone, vascular remodeling, angiogenesis, and neurogenesis [110]. Chemically reactive molecules produced by NO can react with other radicals to form compounds with greater reactivity and toxicity, such as peroxynitrite (ONOO^−^) nitrogen dioxide (NO_2_), and dinitrogen trioxide (N_2_O_3_) [111]. Peroxynitrite has cytotoxic and proinflammatory activity [112]. Superoxide and peroxynitrite are pain mediators. The formation of peroxynitrite after partial nerve injury initiates hyperalgesia and degeneration [113]. Stimulation of cyclic guanosine-monophosphate-cGMP-dependent kinases is also one of the many biological effects of NO. NO activates guanylyl cyclase and increases the synthesis of cyclic guanosine monophosphate, which is important for controlling muscle relaxation and modulating synaptic transmission. Activation of the NO-cGMP-PKG-KATP signaling pathway is involved in the regulation of pain in both the peripheral and central nervous systems. In the peripheral nervous system, this pathway activates the anti-analgesic effects of morphine, while in the central nervous system it increases the expression of μ-opioid receptors (MORs) in the dorsal root ganglia (DRGs). Various studies have demonstrated the effect of the NO-cGMP pathway on the analgesic effect of some drugs for the treatment of neuropathic pain: tramadol [114], clonidine [114], gabapentin [115], and indomethacin [27]. The NO-cGMP pathway has been shown to interact with other signaling pathways [116], such as cholinergic, adrenergic, purinergic, and peptidergic, in the peripheral nervous system, as well as with endocannabinoids [117]. However, this interaction is poorly understood. The NMDA receptor is activated through a voltage-dependent ion channel, which, after activation, increases the content of intracellular Ca^2+^ in the neuron. An increase in intracellular Ca^2+^ activates the constitutive form of NOS, followed by an increase in NO production. NO penetrates into neurons and glia, where it activates sHC and increases the intracellular content of cyclic cGMP. The role of NO and cGMP in the implementation of peripheral nociception remains controversial. NO can contribute to both pro- and antinociceptive effects [118]. The arginine/NO/cGMP activation pathway is antinociceptive in soft tissues (muscles, tendons, ligaments, peripheral nerves) and pronociceptive in intradermal tissues. In nociceptive pain, the effect of NO depends on the stage of inflammation: it is protective in the first hours after inflammation [118] and cytotoxic in the further development of inflammatory pain [119]. Thus, signaling through NO and cGMP can induce pro- or antinociception depending on the different NO concentrations produced [119], on the dose of NOS modulators [120], and localization and stage of the pathological process [118]. A significant increase in the level of NO was found in plasma in patients with chronic pain syndrome compared with healthy people [121]. 

## 5. Conclusions

A comprehensive approach to understanding the effects of NO will help scientists develop new drugs that modulate NO synthesis by targeting *NOSs* to improve the efficacy and safety of pain relief (Table 1). Additionally, it is of interest to clinicians, since the treatment of chronic nociceptive and neuropathic pain in patients with various neurological diseases is a challenging task.

The development of NOS inhibitors may become a future therapy for patients with chronic pain syndromes, which at the present stage of the development of pharmacology is confirmed mainly in animals [122]. However, the effect on NO synthesis and the activity of *NOSs* has been shown in drugs used to treat neurological diseases for many years (COX-selective and non-selective NSAIDs, opioid analgesics, non-opioid analgesics, nitrous oxide, etc.). Selective inhibition of NOS isoforms, especially iNOS and nNOS, is important in the treatment of various types of peripheral pain syndromes. NO is involved in the regulation of COX activity [123] and activates COX with a subsequent increase in the synthesis of prostaglandins. Combinations of NOS inhibitors and COX inhibitors are promising for the treatment of pain syndromes in neurological disorders. It was previously reported that iNOS stimulates COX-2 activity, and thus, provides an effective treatment for neuropathic pain [124]. However, the inconsistency of the available data predetermines further research in the area of analgesic therapy of neuropathic pain through NSAIDs.

## Figures and Tables

**Table 1:** The modulators of NO synthesis for chronic pain syndromes.

Drug	Molecule
Ketorolac	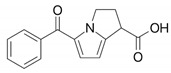
L-NG-Nitroarginine methyl ester (L-NAME)	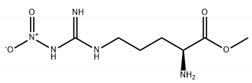
Nanofullerol	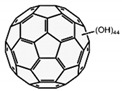
Carboxymethyl lysine (CML)	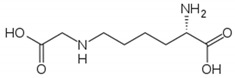
N (ω)-propyl-L-arginine (NPLA)	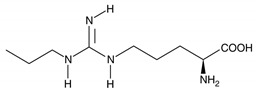
4-Methylaminopyridine	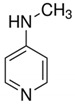
Glycerol trinitrate (GTN)	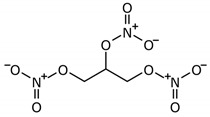
Nitro flurbiprofen(HCT-1026)